# Evaluation of the disinfection efficacy of automated flexible endoscope reprocessors for flexible nasopharyngoscopy: comparison of different disinfectants

**DOI:** 10.3389/fpubh.2026.1817001

**Published:** 2026-06-18

**Authors:** Puyue Zhang, Zhengqi Zhong, Xiaorong Cai, Lihua Yang, Qiuqiong Li, Aitian Tang

**Affiliations:** 1 Central Sterile Supply Department, West China Hospital of Sichuan University, Chengdu, China; 2 Central Sterile Supply Department, West China Xiamen Hospital of Sichuan University, Xiamen, China

**Keywords:** automated flexible endoscope reprocessor, disinfection protocol, flexible nasopharyngoscope, HClO, ortho-phthalaldehyde

## Abstract

**Background:**

Although automated flexible endoscope reprocessors (AER) have been widely used for reprocessing flexible nasopharyngoscopes (FNPs), the disinfection efficacy remains influenced by multiple factors. This study aims to systematically compare the disinfection effectiveness of hypochlorous acid (HClO) and ortho-phthalaldehyde in AER for FNPs, in order to provide evidence for selecting a safe, efficient, and operationally superior disinfection protocol in clinical practice.

**Methods:**

From July to November 2025, this study was conducted in the central sterile supply department of a tertiary hospital. FNPs underwent different disinfection treatments, ortho-phthalaldehyde group with ortho-phthalaldehyde (374 devices) and HClO group with HClO (302 devices). All instruments followed the same standardized cleaning and disinfection protocol and were processed using the same model of AER. The main outcome measures included the number of colony-forming units after disinfection, cleaning qualification rate, drying qualification rate, standard operating procedure compliance rate, operational time consumption, and subjective evaluations by staff regarding their experience with the disinfectants.

**Results:**

The HClO group showed a significantly lower number of colony-forming units after disinfection compared to the ortho-phthalaldehyde group (*P* < 0.05), with a higher proportion achieving zero or low colony levels. Both the cleaning qualification rate and the drying qualification rate were significantly better in the HClO group than in the ortho-phthalaldehyde group (*P* < 0.05). There was no significant difference in standard operating procedure compliance between the two groups (*P* > 0.05), with both maintaining a rate of approximately 99%. In terms of time efficiency, the preparation time and total processing time were significantly shorter in the HClO group than in the ortho-phthalaldehyde group (*P* < 0.05). Staff evaluations indicated that HClO received higher ratings in operational convenience, odor acceptance, and perceived work fatigue.

**Conclusion:**

In AER, the HClO disinfectant demonstrates superior performance in microbial removal efficacy, cleaning, and drying quality compared to ortho-phthalaldehyde. It also offers comprehensive advantages such as simpler operation, shorter processing time, and higher staff acceptance. HClO can serve as an efficient and user-friendly disinfectant option for the automated cleaning and disinfection of FNPs, holding significant potential for broader application.

## 1 Introduction

Flexible nasopharyngoscopy (FNPs) are one of the most widely used, cost-effective, and indispensable diagnostic tools in the clinical practice of otolaryngology (1). They are employed to examine the upper aerodigestive tract, including the pharynx, larynx, and nasal cavity (2). This procedure is relatively quick, straightforward, and has been in use for many years (3). As an invasive technique, FNPs involve direct contact with mucosal surfaces, which may lead to tissue damage, contamination with blood, and exposure to microorganisms (3). Clinically, FNPs are often reused repeatedly within short time intervals (4). These devices have complex internal structures and are made of specialized materials with high precision, making them intolerant to high temperatures and thus creating unique challenges for disinfection and operational handling (5). When FNPs are not thoroughly cleaned and disinfected, opportunistic pathogens residing on the endoscope and its auxiliary equipment can easily spread between patients and healthcare workers (6). Therefore, adequate reprocessing and strengthening the standardized disinfection and management of FNPs are crucial for reducing the risk of pathogen transmission (7).

Traditional FNP cleaning has largely relied on manual washing, while the automated flexible endoscope reprocessor (AER) has made machine-based cleaning of FNPs possible (8). For machine-based chemical disinfection of FNPs, five steps must be completed after a leak test (9). These steps are pre-cleaning, disinfection, rinsing, drying, and storage (9). Disinfection is the key part of machine-based FNP cleaning in reducing pathogen transmission (8). Selecting an environmentally friendly, highly effective, and safe disinfectant is key to minimizing infections associated with FNPs (10). Currently, numerous chemical solutions capable of providing high-level disinfection are available (11). However, due to their distinct characteristics, some disinfectants require pretreatment before use with AER, while others need to be placed in a tank and soaked for a specific contact duration (12). Among these, ortho-phthalaldehyde (OPA) is widely used as a machine disinfectant for FNPs due to its broad-spectrum antimicrobial efficacy (11). This agent acts rapidly, typically within 5–10 min, exhibits minimal irritating odor, demonstrates good compatibility with flexible materials, and provides overall effective disinfection (13). In recent years, hypochlorous acid (HClO) also has gained increasing recognition as a high-level disinfectant (14). It is structurally stable, requires no dilution, mixing, or additives, can be used directly, is easy to store, does not damage endoscopes or accessories, and has a low per-use cost (14). It has been gradually adopted for manual disinfection of FNPs (15). However, the disinfection efficacy of HClO in AER systems remains unclear (16). Based on this, the present study aims to compare the disinfection effectiveness of HClO disinfectant with ortho-phthalaldehyde disinfectant in AER, explore more suitable disinfectants for AER systems, and provide evidence for improving the disinfection efficiency of FNPs and reducing infection risks.

## 2 Methods

### 2.1 Equipment and materials

FNPs (Olympus BF-P180/Q180/MH-533 model); AER (Xinhua brand Rider 60B model); Full-spectrum rapid multi-enzyme cleaner (3M 70508-M model); HClO disinfectant (Jamba medical device, pH 5.7–6.5, containing available chlorine 180–220 mg/L); Ortho-phthalaldehyde disinfectant (Cidex OPA, 0.55%); R2A agar medium and supporting culture supplies; 3M ATP fluorescence detector and surface cleanliness test swabs; 50 ml disposable syringe. Use 0.5% sodium thiosulfate as a neutralizer for HClO, and 0.3% glycine as a neutralizer for ortho-phthalaldehyde, to eliminate the disinfectants during sampling and prevent sustained sterilization.

### 2.2 Study subjects

Between July 1 and September 4, 2025, a total of 374 FNPs were disinfected using ortho-phthalaldehyde as the ortho-phthalaldehyde group. Between September 4 and November 8, 2025, a total of 302 FNPs were disinfected using HClO as the HClO group. Two AER units of the same model were operated in parallel throughout the study period, with each unit using the same disinfectant during each phase. Clinically used FNPs were sequentially and randomly allocated to any available AER unit according to their order of arrival for reprocessing, without any selective intervention during the allocation process. Inclusion criteria: (1) FNPs undergo the complete reprocessing protocol, including pre-cleaning, manual washing, automated rinsing, high-level disinfection, drying, and storage. (2) All steps are performed using an AER and its designated auxiliary equipment. (3) Complete and traceable records for the process and quality control of the cycle are available. This study involved only medical device testing with no human subjects, and formal ethical approval was waived.

Exclusion criteria: (1) Instruments are damaged, under repair, or exhibit structural abnormalities in the insertion tube. (2) Any reprocessing step is interrupted, skipped, or transferred to other equipment. (3) Key quality indicators are missing.

### 2.3 Cleaning and disinfection method

After use, the FNPs undergoes immediate on-site pre-treatment: the operator wipes external contaminants with a gauze moistened with cleaning solution, followed by repeated air and water flushing for at least 10 s. Next, the distal end of the endoscope is immersed in a container with cleaning solution, and suction is activated to draw the solution into the suction channel until it flows fully. Finally, the waterproof cap is secured, and the endoscope is placed in a dedicated container for transport to the disinfection supply center.

**Figure 1:**
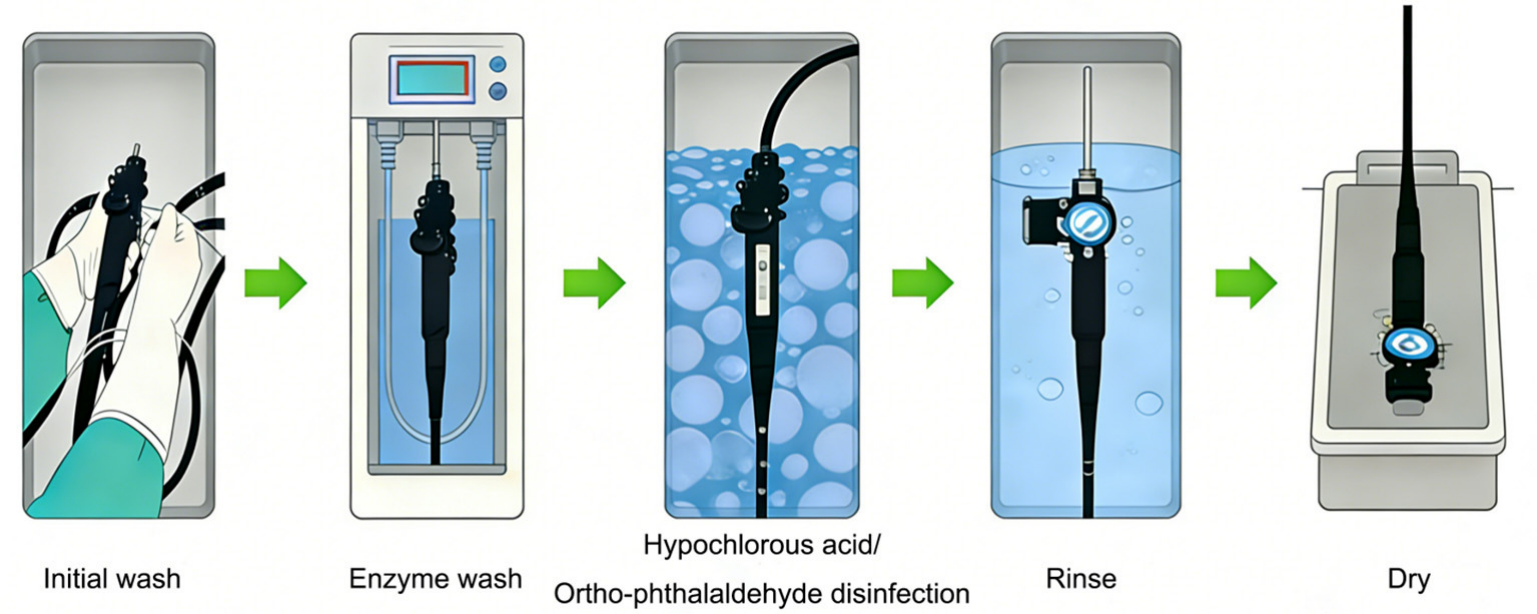
FNPs cleaning process.

The entire cleaning and disinfection process strictly adheres to the operational procedures outlined in the “Technical Specification for Cleaning and Disinfection of Flexible Endoscopes” (WS 507-2016) (17). It is carried out under the joint responsibility of one experienced operator and one monitoring personnel. The procedure begins with a leak test; once no leakage is confirmed, the FNPs is disassembled to its minimal components. These parts are then immersed in multi-enzyme cleaning solution, with both external surfaces and all channels thoroughly brushed. Subsequently, components were rinsed with pressurized water and flushed thoroughly before being dried with an air gun. The FNPs is then connected to an AER. For ortho-phthalaldehyde group, high-level disinfection with the 10 L of 0.55% ortho-phthalaldehyde solution used. For the HClO group, 10 L of HClO medical device disinfectant is added. The machine is then started. The cleaning and disinfection program runs according to the following sequence: (1) Initial wash (including leak testing and manual enzyme cleaning); (2) Followed by enzyme wash; (3) Rinse for 1 min, disinfection; (4) Rinsing using the AER cycle with filtered water; (5) Air drying (Figure 1).

### 2.4 Sampling method

Immediately after the completion of high-level disinfection of FNPs, microbial swab samples were collected. Samples were taken from two sites on each FNP: (1) the optical tip, and (2) the control handle. The swabs were pre-moistened with sterile saline to maximize microbial recovery from the dry surfaces. In addition, microbial swab samples were collected from the outlet port and the inner edge of the cover plate of the AER equipped with ortho-phthalaldehyde disinfectant and HClO disinfectant. Three additional FNPs of the same model were taken. Two of them were artificially contaminated with 0.1 ml 1 × 10^6^ CFU/ml of *Coagulasenegative staphylococcus sp*, and were subjected it to the fully automated cleaning and disinfection process to serve as the positive control group. The remaining one was an unused sterile FNP that underwent no disinfection treatment but was subjected to the same sampling, transport, and inoculation/culturing procedures as the experimental group to serve as the negative control group.

### 2.5 Judgment criteria

The collected 3M surface cleanliness test swab was inserted into the ATP fluorescence detection device and press it all the way to the bottom. The test was performed and the value (RLU) was read. According to the ATP bioluminescence method and the device's reference value, ≤ 200 RLU indicates a successful disinfection. RLU readings were taken after disinfection and after drying.

### 2.6 Reprocessing staff ratings

The ease of use of the disinfectant was evaluated by reprocessing staff using a 5-point Likert scale, where a score of one indicated “very easy to use” and a score of five indicated “quite difficult to use”. The acceptability and strength of the disinfectant odor were assessed by the same staff using the same scale, with response options ranging from “undetectable” to “moderate” on a scale of 1–5 (11). The physical fatigue level of the reprocessing staff was measured using items 1–8 of the Fatigue Scale-14 (FS-14), which reflect physical fatigue. Each item was scored as 1 point for “yes” and 0 points for “no,” with a total possible score of eight points. Higher scores indicated more severe physical fatigue. During the study period, a total of 37 reprocessing staff members participated (18).

### 2.7 Statistical analysis

Data processing and analysis were performed using SPSS 26.0 software (IBM Corporation, Armonk, NY), while data visualization was conducted using Prism 10.0 (GraphPad, San Diego, CA). Normality was assessed using the Shapiro–Wilk test. Normally distributed quantitative data were expressed as mean ± standard deviation (x ± s) and analyzed using independent samples *t*-test for between-group comparisons. Categorical data were expressed as number (percentage) [*n* (%)] and analyzed using χ^2^ test. Categorical variables were analyzed using the Mann-Whitney *U*-test. *P* < 0.05 was considered statistically significant.

## 3 Results

### 3.1 Comparison of FNPs basic characteristics

There were no statistically significant differences in the clinical setting, type of FNPs, or disinfection temperature between the two groups of instruments (*P* > 0.05). The FNPs in the HClO group had a longer service life compared to those in the ortho-phthalaldehyde group (*P* < 0.05) (Table 1).

**Table 1:** Comparison of FNPs basic characteristics.

Characteristics	Ortho-phthalaldehyde group (*n* = 374)	HClO group (*n* = 302)	*t*/χ^2^	*P*
**Clinical setting**, ***n*** **(%)**				
Outpatients	53 (14.17)	32 (10.60)	1.943	0.163
Inpatients	321 (85.83)	270 (89.40)		
**Type of FNPs**, ***n*** **(%)**				
Adult FNPs	352 (94.12)	281 (93.05)	0.322	0.570
Pediatric FNPs	22 (5.88)	21 (6.95)	–	–
FNPs service life, years ($\bar{x}\;\pm \;s$)	5.03 ± 2.71	5.48 ± 3.02	−2.039	0.042
Disinfection temperature, °C ($\bar{x}\;\pm \;s$)	17.15 ± 0.28	17.19 ± 0.31	−1.760	0.079

### 3.2 Evaluation of FNPs disinfection efficacy

Statistical analysis of colony forming units (CFUs) before and after disinfection revealed no significant difference in the number of CFUs between the two groups prior to disinfection (*P* > 0.05). However, a statistically significant difference was observed between the two groups after disinfection (*P* < 0.05), with the HClO group showing a higher number of cases achieving 0 CFU and 1–50 CFUs compared to the ortho-phthalaldehyde group. In the ortho-phthalaldehyde group, the number of colonies decreased on 358 FNPs after disinfection, while in the HClO group, the number of colonies decreased on 299 FNPs after disinfection (Table 2). The positive control showed a significant decrease in CFU counts after disinfection. Additionally, 10 FNPs from each group were randomly selected for microbial culture analysis. Swab samples were collected from the inner rim of the AER, the optic tip of the FNPs, and the handle of the FNPs and sent for testing (Table 3). After treatment with ortho-phthalaldehyde disinfectant and HClO disinfectant, no positive cultures were detected from the inner rim of the AER in either group. Similarly, no positive cultures were detected from the handle of the FNPs treated with HClO disinfectant. However, among the swabs from the optic tip of FNPs treated with ortho-phthalaldehyde disinfectant, four samples grew *Coagulasenegative staphylococcus sp*, while the remaining samples showed no growth. From the handle of FNPs treated with ortho-phthalaldehyde disinfectant, three samples tested positive for *Stenotrophomonas maltophilia*, with the remaining samples showing no growth. Furthermore, among the swabs from the optic tip of FNPs treated with HClO disinfectant, three samples grew *Neisseria*, while the remaining samples showed no growth.

**Table 2:** Colony forming units (CFUs) before and after disinfection in both groups.

Characteristics	Ortho-phthalaldehyde group (*n* = 374)	HClO group (*n* = 302)	*Z*	*P*
**Before disinfection**				
**Number of CFUs**, ***n*** **(%)**				
0 CFU	0 (0.00)	0 (0.00)	−1.524	0.128
1–50 CFUs	42 (11.23)	49 (16.23)		
51–500 CFUs	173 (46.26)	136 (45.03)		
>500 CFUs	159 (42.51)	117 (38.74)		
Before disinfection-positive control group	>500 CFUs	>500 CFUs		
**After disinfection**				
**Number of CFUs**, ***n*** **(%)**				
0 CFU	269 (71.93)	242 (80.13)	11.278	0.010
1–50 CFUs	102 (27.27)	58 (19.21)		
51–500 CFUs	2 (0.53)	1 (0.33)		
>500 CFUs	1 (0.27)	1 (0.33)		
After disinfection-positive control group	13 CFUs	10 CFUs		
Number of FNPs with colonies cleared, n	358	299		
Number of FNPs with colonies increased, *n*	0	0		

**Table 3:** Residual microorganisms at different sites with different disinfectants.

Disinfection	Sample site	Organism grown	Relevance
Ortho-phthalaldehyde + positive control group	Inner rim of the AER machine lid	No growth	–
	Optic tip of FNPs	Light growth of *Coagulasenegative staphylococcus sp*	Common low-pathogenicity commensals
	Handle of FNPs	Light growth of *Coagulasenegative staphylococcus sp*	Common low-pathogenicity commensals
HClO + positive control group	Inner rim of the AER machine lid	No growth	–
	Optic tip of FNPs	Light growth of *Coagulasenegative staphylococcus sp*	Common low-pathogenicity commensals
	Handle of FNPs	No growth	
Negative control group	Inner rim of the AER machine lid	No growth	
	Optic tip of FNPs	No growth	–
	Handle of FNPs	No growth	–
Ortho-phthalaldehyde group	Inner rim of the AER machine lid	No growth	–
	Optic tip of FNPs	Light growth of *Coagulasenegative staphylococcus sp* and *Neisseria*	Low-pathogenicity skin commensals, which may be attributed to inadequate exposure of disinfectant at that specific site of the FNPs. Common respiratory flora, tolerant to low temperatures, potentially due to lower temperatures.
	Handle of FNPs	Light growth of *Stenotrophomonas maltophilia*	Low-pathogenicity environmental microorganism, which is intrinsically resistant to antibiotics.
HClO group	Inner rim of the AER machine lid	No growth	–
	Optic tip of FNPs	Light growth of *Neisseria*	Common respiratory flora, tolerant to low temperatures, potentially due to lower temperatures.
	Handle of FNPs	No growth	–

Further evaluation of disinfection time revealed no statistically significant difference in disinfection duration between the two groups (*P* > 0.05). However, the preparation time and total process time were significantly longer in the ortho-phthalaldehyde group compared to the HClO group (*P* < 0.05) (Table 4).

**Table 4:** Comparison of time consumption between the two groups.

Characteristics	Ortho-phthalaldehyde group (*n* = 374)	HClO group (*n* = 302)	*t*	*P*
Disinfectant time, minutes ($\bar{x}\;\pm \;s$)	11.22 ± 1.58	10.99 ± 1.62	1.860	0.063
Preparation time, minutes ($\bar{x}\;\pm \;s$)	17.95 ± 3.12	14.34 ± 4.51	12.267	< 0.001
Total time, minutes ($\bar{x}\;\pm \;s$)	29.06 ± 6.98	23.07 ± 6.15	11.692	< 0.001

The cleaning qualification rate and drying qualification rate in the HClO group were significantly higher than those in the ortho-phthalaldehyde group (*P* < 0.05). The ATP fluorescence detection values in the HClO group after cleaning and after drying were both significantly higher than those in the ortho-phthalaldehyde group (*P* < 0.05). The differences in pass rates after cleaning and drying between the two groups were not significant (*P* > 0.05). There was no statistically significant difference in the compliance level of standard operating procedures (SOP) between the two groups (*P* > 0.05) (Table 5).

**Table 5:** Comparison of disinfection efficacy between the two groups.

Characteristics	Ortho-phthalaldehyde group (*n* = 374)	HClO group (*n* = 302)	*t*/χ^2^	*P*
**Overall cleaning qualification**, ***n*** **(%)**				
Qualified	342 (91.44)	288 (95.36)	4.049	0.044
Unqualified	32 (8.56)	14 (4.64)		
ATP fluorescence detection, RLU ($\bar{x}\;\pm \;s$)	51.43 ± 8.75	44.68 ± 10.51	9.111	< 0.001
**Drying qualification**, ***n*** **(%)**				
Qualified	357 (95.45)	298 (98.68)	5.750	0.016
Unqualified	17 (4.55)	4 (1.32)		
ATP fluorescence detection, RLU ($\bar{x}\;\pm \;s$)	42.61 ± 10.88	37.26 ± 13.51	5.703	< 0.001
Comparison of cleaning and drying pass rates, *n* (%)	342	288	–	–
	325	280	–	–
χ^2^	4.001	1.890	–	–
15.6-7.2,-1.3498pt*P*	0.051	0.169	–	–
**SOP compliance**, ***n*** **(%)**				
Qualified	370 (98.93)	299 (99.01)	0.009	0.923
Unqualified	4 (1.07)	3 (0.99)		

### 3.3 Reprocessing staff evaluation

**Figure 2:**
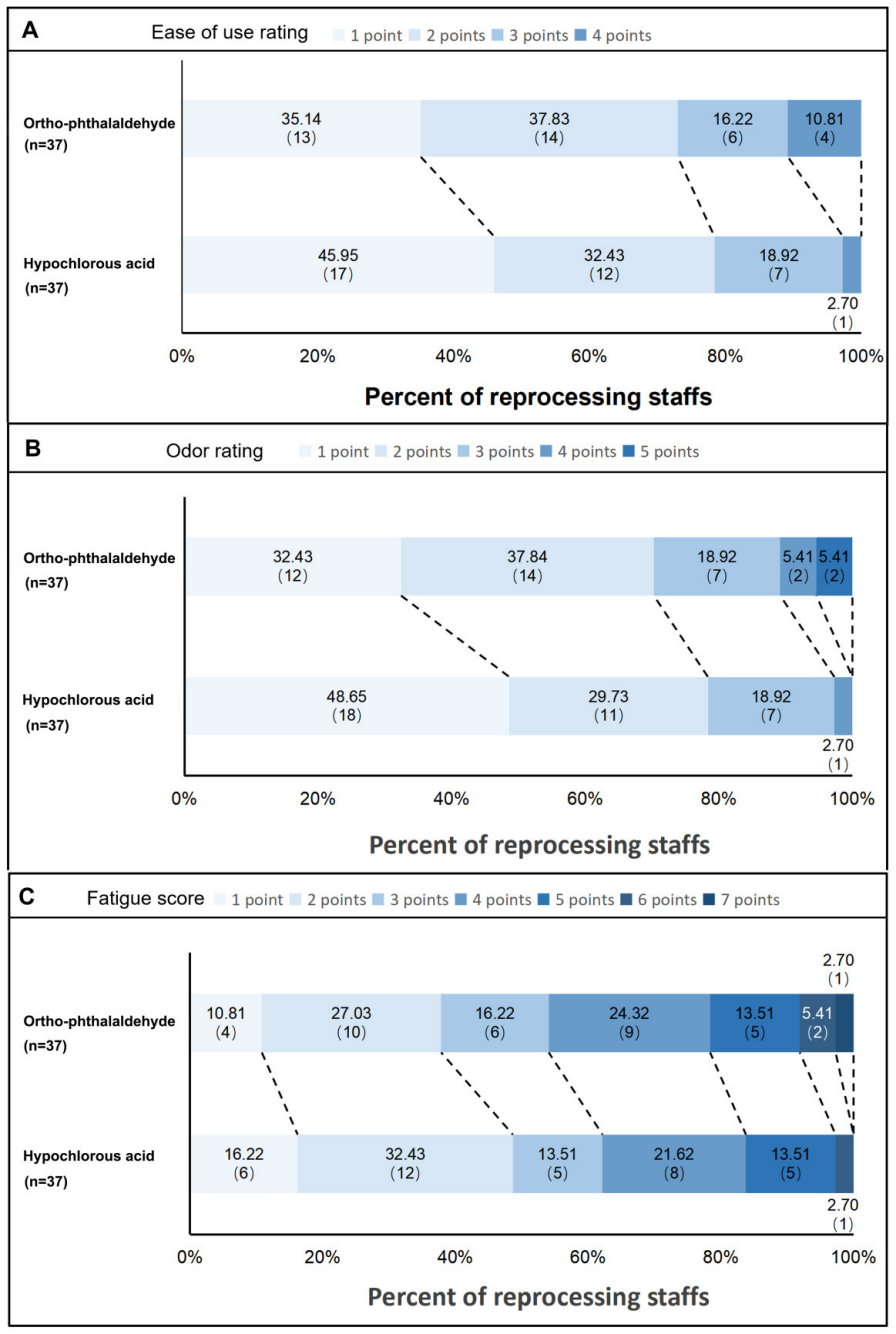
Reprocessing staff ratings. **(A)** Ease of use rating. **(B)** Odor rating. **(C)** Fatigue score.

In the evaluation of ease of use by reprocessing staff for the HClO group, nearly half of the respondents considered it convenient to use, which was higher compared to the feedback for ortho-phthalaldehyde group (Figure 2A). Regarding odor assessment, nearly half of the staff reported that hypochlorous acid had a relatively mild odor, while only 32.43% of respondents perceived ortho-phthalaldehyde as having a mild odor. Additionally, 5.41% of the staff found the odor of ortho-phthalaldehyde intolerable (Figure 2B). Assessments of fatigue levels among reprocessing staff also varied between the two disinfectants, with 2.7% of respondents indicating that the disinfection process using ortho-phthalaldehyde was highly fatiguing (Figure 2C).

## 4 Discussion

Although AER have been widely used for disinfecting FNPs, their effectiveness remains influenced by multiple factors, particularly due to a lack of comprehensive comparative studies on the actual performance of different chemical disinfectants within AER (19). Ortho-phthalaldehyde disinfectants are commonly used, but their need for pretreatment and their characteristic irritating odor indicate an urgent need for better alternatives (20). HClO has shown promising results in manual disinfection (14). However, its applicability, stability, and final disinfection efficacy within AER remain unclear (14). Therefore, a scientific comparison of the comprehensive performance of these two disinfectants in the AER cleaning and disinfection process holds direct practical significance for optimizing the disinfection strategy for FNPs and enhancing infection control standards.

This study found that the HClO group showed superior performance in terms of CFU count after disinfection, suggesting that hypochlorous acid may have stronger clearance capabilities for microorganisms on the surface and lumens of FNPs (21). Furthermore, microbial culture analysis revealed that the ortho-phthalaldehyde group still harbored small amounts of low-pathogenicity microorganisms at the lens tip and handle, which may be attributed to inadequate contact time or poor penetration of ortho-phthalaldehyde in complex structural areas (22). In contrast, the HClO group detected only common respiratory colonizing bacteria at the lens tip. This difference may be related to the physicochemical properties and mechanism of action of the active ingredients in HClO (23). The active components in HClO possess stronger permeability and diffusivity, allowing them to effectively penetrate biofilm structures and cover complex instrument surfaces (9). Their oxidative mechanism rapidly disrupts microbial cellular structures, thereby achieving more thorough disinfection (1). Previous studies have also indicated that disinfectants with mechanisms similar to those of HClO exhibit good clearance effects on biofilms and some drug-resistant pathogens (23). The findings of this study further support the potential advantages of HClO in AER.

Regarding time consumption, there was no significant difference in the required time for the disinfection phase between the two groups, indicating that both disinfectants can complete the preset chemical action cycles during AER operation. However, the preparation time and total operational time for the ortho-phthalaldehyde group were significantly longer than those for the HClO group. This difference primarily stems from the necessary pre-treatment steps required for ortho-phthalaldehyde, such as solution preparation, concentration verification, and instrument pre-soaking, which increase operational complexity and manual labor time (24). In contrast, HClO, as a ready-to-use disinfectant, can be directly injected into the washing and disinfection machine without dilution or activation, significantly simplifying the preparation process and reducing the time occupied by non-disinfection procedures (15). This further suggests that while pursuing disinfection efficacy, operational convenience and process efficiency are also important considerations for optimizing FNP reprocessing protocols (25).

The overall cleaning qualification rate and drying qualification rate in the HClO group were significantly higher than those in the ortho-phthalaldehyde group. This difference may be attributed to the characteristics of HClO as a ready-to-use disinfectant. Its no-need-for-preparation and high stability reduce variable factors in operational steps, facilitating the complete execution of cleaning procedures (26). At the same time, the properties of its solution may have a synergistic effect on the subsequent drying process, suggesting that the physicochemical characteristics of the disinfectant itself may directly influence the wetting state and drying efficiency of the instrument surface after cleaning (27). It is worth noting that the compliance with standardized operating procedures in both groups remained at a high level with no statistically significant difference, indicating that under the current standardized operational framework, both disinfectants can achieve the execution of standardized processes (28). This suggests that when selecting disinfectants, attention should also be paid to their compatibility with existing standardized processes and their practical impact on key quality aspects (11).

The comprehensive evaluation by reprocessing staff showed that HClO received higher ratings than ortho-phthalaldehyde in terms of operational ease of use, odor acceptance, and perception of work fatigue. This difference is primarily attributed to HClO's characteristics as a ready-to-use disinfectant. Its simplified operation, which requires no preparation or activation, reduces work steps and waiting time, thereby lowering operational complexity (29). At the same time, its relatively mild odor improves workplace comfort and may indirectly reduce the psychological burden and physical fatigue of the operators (29). This suggests that in the selection of disinfectants, aside from microbiological efficacy, operational friendliness and staff acceptance are also important factors influencing the sustainability of overall reprocessing quality (11).

However, this study still has certain limitations. The research primarily focused on process indicators such as cleaning qualification rates and drying effectiveness, and did not extend to tracking actual clinical outcomes such as infection rates and patient adverse events associated with the use of endoscopes reprocessed with the two disinfectants. In the future, it will be necessary to conduct multicenter randomized controlled trials, combined with long-term dynamic microbial monitoring, evaluation of endoscope biofilm burden, and prospective tracking of patient infection outcomes, to further verify the long-term efficacy and safety differences of the two disinfectants in real clinical environments. In addition, the two stages of this study were in summer and autumn, respectively, during which the spectrum of common viruses and bacteria in the upper respiratory tract may have seasonal differences. Although, in order to control this bias, we verified the performance stability of the disinfection equipment across the two stages through a positive control group, future cross-seasonal, prospective studies are still needed to more comprehensively evaluate the stability of disinfectants in real clinical settings. Due to the obvious differences in appearance, odor, and application method between the two disinfectants, the reprocessing staff involved in the evaluation could not be blinded. This lack of blinding may have had some influence on the staff's subjective evaluations. Although the objective outcome measures were not affected by the absence of blinding, future studies should still attempt to incorporate blinding whenever possible to minimize bias in subjective assessments.

## 5 Conclusion

In AER systems, the HClO disinfectant demonstrated superior performance in microbial removal efficacy, cleaning and drying qualification rates compared to ortho-phthalaldehyde, along with comprehensive advantages such as simpler operation, shorter processing time, and higher staff acceptance. These findings suggest that HClO possesses the potential to serve as the preferred disinfectant for FNPs in AER-based disinfection protocols.

## Data Availability

The raw data supporting the conclusions of this article will be made available by the authors, without undue reservation.
